# Development of a High‐Sensitivity Electrochemical Biosensor for Domoic Acid and Its Cellular Impact on Human Stem and Neuron‐Like Cells

**DOI:** 10.1002/fsn3.71799

**Published:** 2026-04-27

**Authors:** Emilia Qomi Ekenel, Yucel Koc, Burcugul Altug, Merve Nur Soykan, Bahar Demir Cevizlidere, Sibel Gunes Bagis, Onur Uysal, Tugba Semerci Sevimli, Murat Sevimli, Ayla Eker Sariboyaci, Huseyin Avci

**Affiliations:** ^1^ Cellular Therapy and Stem Cell Production Application and Research Centre, ESTEM Eskisehir Osmangazi University Eskisehir Turkey; ^2^ Department of Chemical Engineering Eskisehir Osmangazi University Eskisehir Turkey; ^3^ Department of Genetics, Faculty of Veterinary Medicine Dokuz Eylül University İzmir Turkey; ^4^ Department of Stem Cell, Institute of Health Sciences Eskisehir Osmangazi University Eskisehir Turkey; ^5^ Department of Histology and Embryology, Faculty of Medicine Eskisehir Osmangazi University Eskisehir Turkey; ^6^ Department of Metallurgical and Materials Engineering Eskisehir Osmangazi University Eskisehir Turkey; ^7^ Translational Medicine Research and Clinical Center (TATUM) Eskişehir Osmangazi University Eskisehir Turkey

**Keywords:** cytotoxicity assessment, domoic acid, electrochemical biosensor, human amniotic fluid mesenchymal stem cells (hAF‐MSCs), human neuroblastoma cells (SH‐SY5Y)

## Abstract

Domoic acid (DA) is an important environmental neurotoxin produced by certain types of marine algae and can accumulate in seafood such as anchovies, sardines, and mussels, which are considered healthy and widely consumed globally. One of the most important issues to consider is taking necessary measures to prevent water and marine pollution and conducting proper safety controls before consuming seafood. This study aimed to develop a high‐sensitivity electrochemical biosensor system for detecting DA in seafood, aiding marine pollution monitoring. Additionally, the cytotoxicity of DA at the highest Food & Drug Administration (FDA)‐permitted concentration and the concentrations determined in this study was examined on human cells. Gene expression related to cytotoxic response, DNA repair, and neurotoxicity was analyzed using amniotic fluid‐derived mesenchymal stem cells (hAF‐MSCs) and the human neuroblastoma cell line (SH‐SY5Y) as neuron‐like cell models. DA did not significantly affect the viability of hAF‐MSCs or SH‐SY5Y cells at 48‐h exposure but slightly reduced viability at 100 μg/mL compared to the control group. DA activated the glutamate receptor in hAF‐MSCs but not in SH‐SY5Y cells. It also reduced DNA repair capacity in hAF‐MSCs, though effects on viability and proliferation likely did not manifest within 48 h. There was no change in gene expression at 75 ng/mL of DA, which indicated the tolerable daily intake set by the FDA. The SH‐SY5Y cells exhibited resistance against DA, indicating enhanced their DNA repair to prevent themselves against apoptosis and cell death. It is also supported by the upregulation of DNA repair‐related genes and the membrane transporter gene ABCG2.

## Graphical abstract

Integrated workflow of domoic acid (DA) detection in seafood using a high‐sensitivity electrochemical biosensor and subsequent evaluation of DA‐induced cellular responses. (A) DA monitoring in seafood: Marine samples (European anchovy and mussels from the Marmara and Black Sea) were collected, extracted using a 1:1 methanol/water mixture, and analyzed via a screen‐printed gold electrode (SPGE)‐based electrochemical impedance spectroscopy (EIS) biosensor. The biosensor detected trace levels of DA (0.589635 ng/mL) in Marmara mussels, while no DA was found in anchovy samples. (B) Cellular toxicity analysis: Human amniotic fluid‐derived mesenchymal stem cells (hAF‐MSCs) and SH‐SY5Y neuroblastoma cells were treated with environmentally and regulatorily relevant DA concentrations (0.5 ng/mL to 100 μg/mL). Cytotoxicity and gene expression analyses were performed via MTT assay and qRT‐PCR. Genes analyzed include DNA repair (e.g., MRE11, XRCC4), apoptotic/antiapoptotic markers (e.g., BCL2, TNFA), and glutamate receptor subunit GRIA2. Created in BioRender. Avci, H. (2025) https://BioRender.com/j88o058.

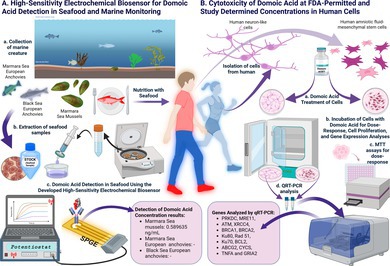

## 1 Introduction

Foodborne diseases are associated with pathogens, toxins, and other pollutants that have contaminated food, which are a serious threat to human health. Three commodities for aquatic animals, fish, crustaceans, and mollusks, were defined for foodborne outbreak investigations by the “World Center for Disease Control and Prevention” report. In 2016, fish products were under the single food categories associated with the most outbreaks (Centers for Disease Control and Prevention [CDC] 2017).

Fish and shellfish can have a high in toxic substances of methylmercury and domoic acid (DA). DA is a neurotoxin, an analog of kainic acid, produced by algae of the genus *Pseudo‐nitzschia* and *Nitzschia navis‐varingica* in nature (Tenorio et al. 2021). Through bioaccumulation, it can reach the food chain and then reach people. When DA and its isomers are consumed above a certain amount, they can cause short‐term or permanent memory loss, poisoning can happen, nausea, vomiting, diarrhea, abdominal cramps, dizziness, headache, disorientation, seizures, coma and eventually death (Washington State Department of Health, n.d.). For these reasons, DA is recognized as an important environmental neurotoxin that poses a global risk to the health and safety of both humans and wildlife (Lefebvre and Robertson 2010). Nowadays, the danger of DA is increasing rapidly in seafood such as anchovies, sardines, and mussels which are thought to be healthy and consumed by many people worldwide. The maximum level of DA consumption is limited by the United States Food and Drug Administration (FDA 1993), which makes necessary measures to prevent water and marine pollution in addition to control before consuming seafood. Traditional detection methods, which require some advanced tools and trained personnel, are widely used to identify bacterial pathogens and toxins. However, these methods are time‐consuming and laborious. Electrochemical biosensors, particularly when integrated with advanced techniques such as electrochemical impedance spectroscopy (EIS), have shown great promise in surface analysis applications. One notable advancement is the modification of electrode surfaces using nanomaterials to enhance sensitivity and specificity (Li et al. 2025; Zhang et al. 2023). Therefore, one of the aims of this study is to develop an electrochemical biosensor system with high sensitivity for the investigation of domoic acid in seafood, and then to investigate the toxicity of DA at various concentrations on living cells with the gene expression of genes related to cellular response against a cytotoxic agent. Furthermore, the DNA repair mechanism is investigated. As DA crosses the placenta and it has been associated with neuronal damage in fetuses (Shum et al. 2020), amniotic fluid‐derived mesenchymal stem cells (hAF‐MSCs) from fetal period tissues (Washington Department of Fish and Wildlife, n.d.) and the human neuroblastoma cell line of SH‐SY5Y representing neuron‐like cells were selected as the experimental model for this study. DNA repair is an important mechanism that protects the cell which undergoes a DNA damage pathway that can start via a mutation and sometimes can end with cancer. Genetic instability is the major cause of cancer. Mistakes in DNA repair are also genetic instability. The majority of cancers are due to unrepaired DNA damage. DA concentrations were determined based on the concentrations reported to have neurotoxic effects obtained from the literature (Shum et al. 2020), the maximum concentration determined by the Washington Department of Fish and Wildlife (n.d.), the tolerable daily intake (TDI) of domoic acid for humans (Kumar et al. 2009), and the concentration which was detected by our developed biosensor.

Briefly in this study, we aimed to (i) develop a highly sensitive electrochemical biosensor for detecting trace levels of domoic acid (DA) in seafood and (ii) investigate the cytotoxic, neurotoxic, and genotoxic impacts of DA on human amniotic fluid‐derived mesenchymal stem cells (hAF‐MSCs) and human neuroblastoma SH‐SY5Y cells, respectively. Although the biosensor development and cellular effect analysis of domoic acid may appear as two distinct sections, they are tightly connected in this study. The determined DA concentration in seafood was directly used to evaluate its relevance to human exposure by in vitro testing. Therefore, the integration is not arbitrary but designed to bridge environmental detection with cellular toxicity in a single workflow.

## 2 Material and Methods

### 2.1 Electrochemical Experimental Setup

Ensuring constant ambient conditions is essential for electrochemical experiments to achieve optimal sensitivity, precision, and consistency. The Gamry Reference 3000 Potentiostat/Galvanostat/ZRA instrument was used to perform the EIS study. Echem Analyst was used to evaluate the acquired data. Following the immobilization of the screen‐printed gold electrodes (SPGEs; DropSens, Oviedo, Spain), the electrochemical behavior of the domoic acid detection was assessed using a redox probe, which was made up of a mixed solution of 1 mM potassium ferricyanide (K_3_[Fe(CN)_6_]) and K_4_Fe(CN)_6_ (Sigma‐Aldrich, Merck KGaA, Darmstadt, Germany) in 1 mM phosphate‐buffered saline (PBS). With a perturbation signal of 10 mV, impedance measurements were performed under open circuit potential (OCP). To reduce environmental electrical interference, a Gamry Faraday cage was employed. The DRP‐220AT type SPGEs were purchased from DropSens, as were the matching connections. SPGEs are comprised of working (WE), counter (CE), and reference electrode (RE) systems made of gold, gold, and silver, respectively. The gold working electrode had a surface area of approximately 0.1256 cm^2^ (Koç et al. 2021a, 2021b; Koç and Avci 2024).

### 2.2 Study Area and Investigated Species

**FIGURE 1:**
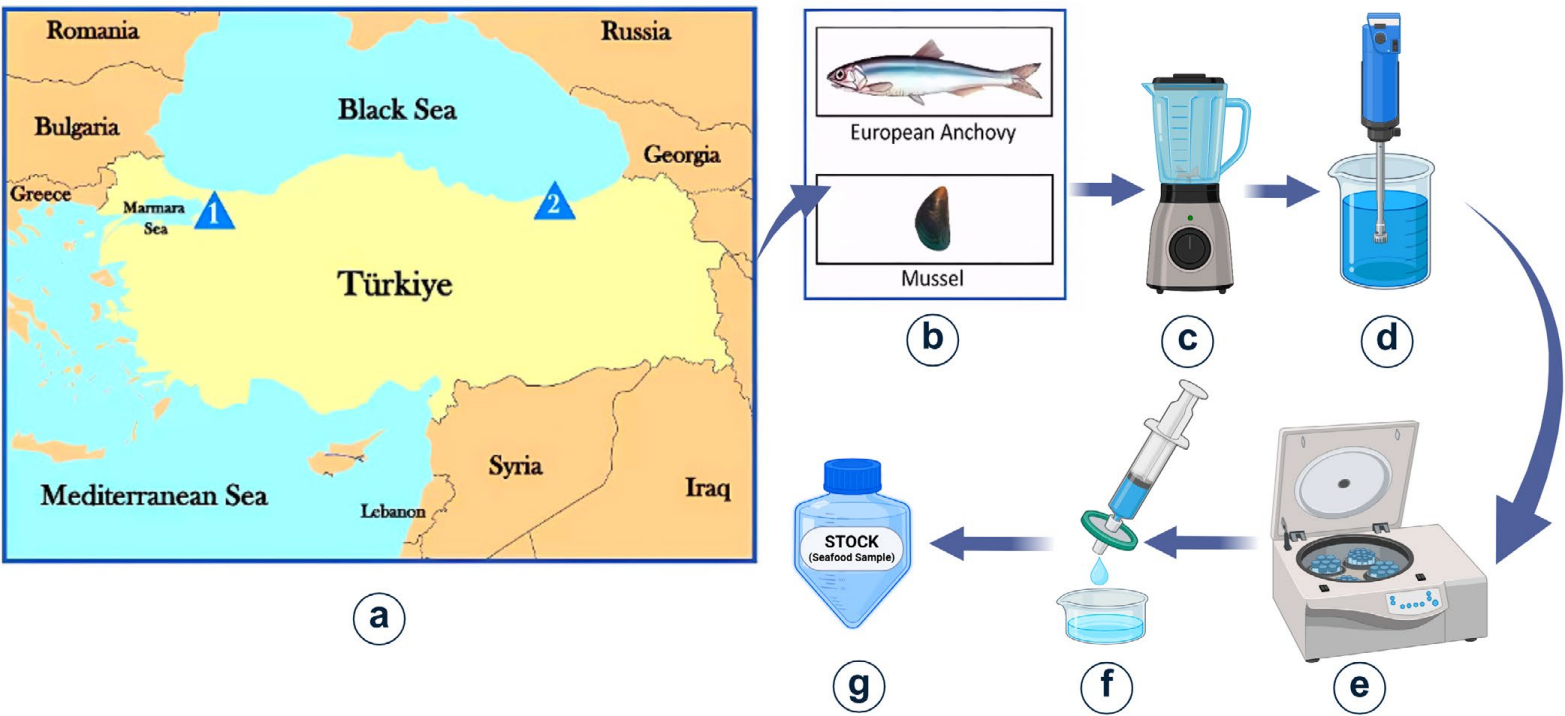
(a) Map of Turkey showing the coastal sampling stations (Triangle 1: Kocaeli, Marmara Sea coast; Triangle 2: Trabzon, Black Sea coast). (b) Collected seafood samples: European anchovy ( *Engraulis encrasicolus* ) and mussel ( *Mytilus galloprovincialis* ). (c) Sample mincing and blending. (d) Homogenization in methanol/water (1:1, v/v). (e) Centrifugation at 4000 rpm for 20 min. (f) Filtration of the supernatant using a 0.22 μm methanol‐compatible filter. (g) Storage of extracted samples in sterile tubes at 4°C. Each extraction step was conducted under sterile conditions. All seafood extracts were prepared using a standardized protocol involving homogenization in 1:1 methanol/water (v/v), centrifugation, filtration through 0.22 μm membrane filters, and storage at 4°C. The filtered extracts were later applied to the biosensor surface and incubated for 30 min. Each extraction was performed in triplicate to ensure reproducibility and consistency in biosensor response. Created in BioRender. Avci, H. (2025) https://BioRender.com/xfc9ssj.

This study investigates the presence of domoic acid in marine creatures collected from various regions along the Marmara and Black Sea coasts in Turkey. Marine creatures were collected from Turkey's Marmara Sea and Black Sea coastal cities of Kocaeli and Trabzon during the winter season January and February 2020 with the permission from the Republic of Turkey Ministry of Agriculture and Forestry, General Directorate of Fisheries and Aquaculture (67852565‐140.03.03‐E.1415263, 08.06.2017). European anchovies ( *Engraulis encrasicolus* ) and mussels ( *Mytilus galloprovincialis* ) were collected directly from the sea (Washington Department of Fish and Wildlife, n.d.). The species investigated include European anchovies and mussels from the Marmara region, and only European anchovies from the Black Sea region. In Figure 1a, European anchovies and mussels obtained from Kocaeli city in Turkey (Triangle‐1) province, province along the Marmara Sea coast, were obtained from the location at coordinates 40°45′33″ N and 29°55′22″ E. Another sample is the European anchovy, obtained from the province of Trabzon city in Turkey (Triangle‐2) on the coast of the Black Sea, at 41°03′11″ N and 39°32′21″ E coordinates.

### 2.3 Seafood Extraction Protocol

Seafood samples were prepared (Figure 1b–e) following ethical approval (Eskisehir Osmangazi University Animal Experiments Local Ethics Committee, 612/2017, 19.07.2017). Whole anchovies and mussels were cut into small pieces and homogenized using a blender. Four grams of each homogenate were mixed with 16 mL of methanol/water (1:1, v/v, Merck KGaA/ultrapure, Merck Millipore, Burlington, MA, USA) and further homogenized for at least 3 min. The resulting mixture was centrifuged at 4000 rpm for 20 min. The supernatant was filtered through a 0.22 μm methanol‐compatible syringe filter (Minisart RC 25, Sartorius Stedim Biotech GmbH, Göttingen, Germany) and stored in sterile tubes at 4°C until further analysis. The filtered extracts were later applied to the biosensor surface and incubated for 30 min. Each extraction was performed in triplicate to ensure reproducibility and consistency in biosensor response. Domoic acid (DA) detection was performed using SPGEs functionalized under previously optimized conditions (Washington Department of Fish and Wildlife, n.d.).

### 2.4 Bio‐Immobilization Process of Anti‐Domoic Acid (DA) Antibody

**FIGURE 2:**
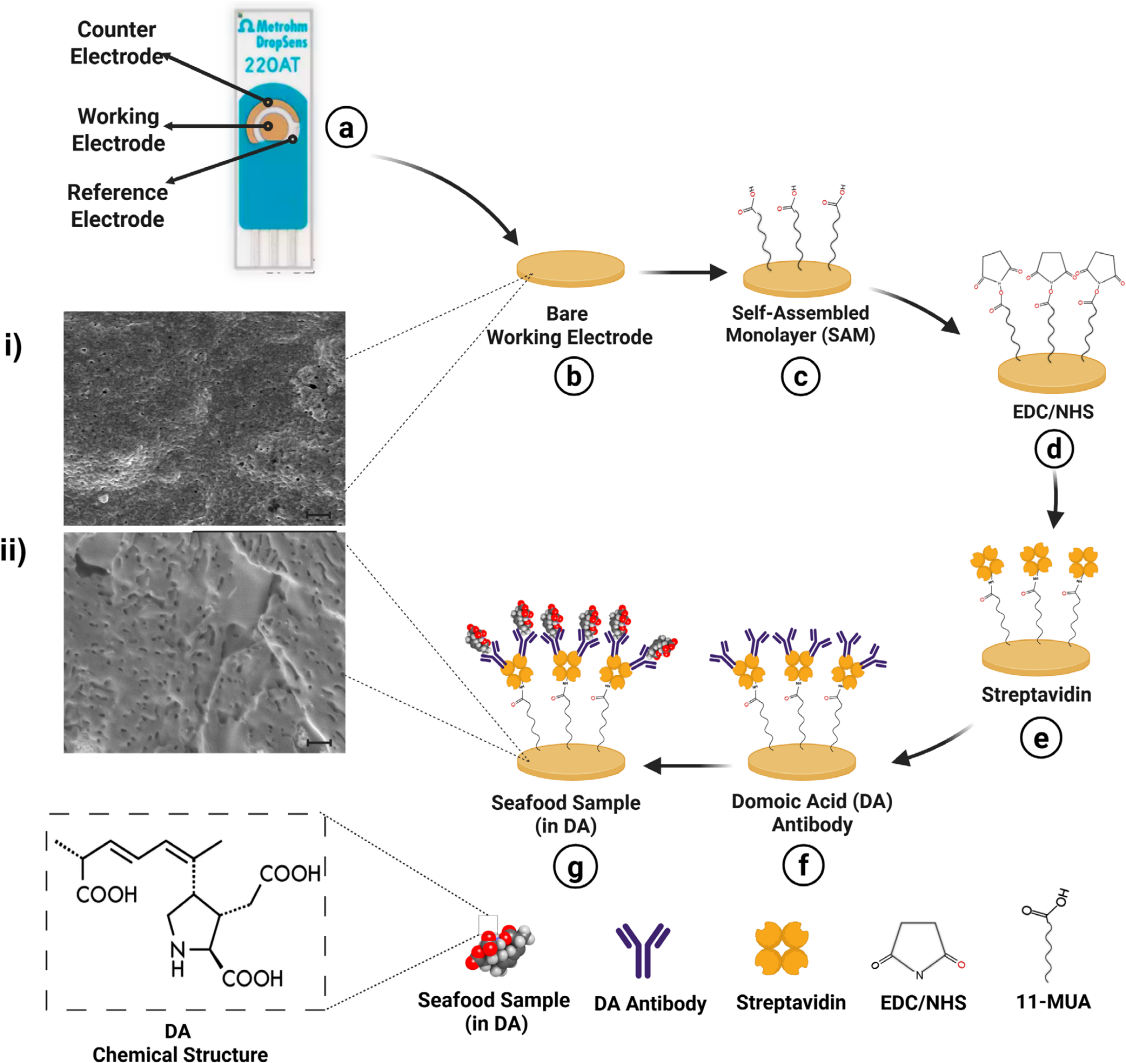
A schematic illustration of the bio‐immobilization steps for screen‐printed gold electrodes (SPGEs) (a–g). (a) Bare SPGE, (b) formation of self‐assembled monolayer (SAM) with 11‐mercaptoundecanoic acid, (c) activation with EDC/NHS chemistry, (d) immobilization of streptavidin, (e) conjugation with anti‐domoic acid monoclonal antibody (mAb), (f) application of seafood extract samples, and (g) final biosensor configuration after DA binding process.. All incubations were performed at room temperature unless otherwise stated. The antibody immobilization and sample binding steps were conducted for 1 h each. EIS was performed using 1 mM [Fe(CN)_6_]^3−^/^4−^ in 1 mM PBS, with 10 mV amplitude at open circuit potential. The scanning electron microscopy (SEM) images (i, ii) display: (i) the surface morphology of the bare SPGE, and (ii) the SPGE surface after antibody immobilization. Scale bars in SEM images represent 3 μm. Created in BioRender. Avci, H. (2025) https://BioRender.com/c5e492r.

The bio‐functionalization of SPGEs followed a stepwise immobilization protocol (Figure 2a–g). First, a 10 mM solution of 11‐mercaptoundecanoic acid (11‐MUA) in ethanol was incubated on the gold electrode for 1 h to form a self‐assembled monolayer (SAM), introducing carboxylic groups. After ethanol and PBS washes, the surface was activated with a 1:1 mixture of 50 mM EDC and NHS in citric acid buffer for 30 min. Streptavidin (1.25 μg/mL) was then immobilized onto the activated surface for 1 h, followed by PBS washing. Next, a monoclonal anti‐domoic acid (DA) antibody (1.25 μg/mL in PBS) was applied for 1 h. After removing unbound molecules, seafood extracts or DA‐containing solutions were incubated on the functionalized electrodes for 30 min. Electrochemical impedance spectroscopy (EIS) was performed in 1 mM [Fe(CN)_6_]^3−/4−^ in 1 mM PBS with a 10 mV amplitude under open circuit potential. After these immobilizations, the system was tested by adding solutions containing DA (1 ng/mL DA in cell culture media [CCM] and 1 ng/mL DA [in methanol]) to the electrode surface. Nyquist plots were modeled using the Randles equivalent circuit via Gamry Echem Analyst software. All incubations during antibody immobilization and sample application were performed at room temperature unless otherwise stated. All impedance measurements were repeated in three independent experiments. The standard deviation among the replicates was found to be < 5%, indicating excellent reproducibility.

### 2.5 Electrochemical Impedance Spectroscopy (EIS) Measurements

EIS, an electroanalytical technique, was employed to detect DA in seafood extracts with high sensitivity. EIS allows real‐time, noninvasive, and precise monitoring of molecular interactions at the electrode/electrolyte interface by applying a small‐amplitude sinusoidal voltage over a range of frequencies and measuring the system's impedance response (Vivier and Orazem 2022; Wang et al. 2021).

The impedance (*Z*) is a complex function of frequency (*ω*) and is mathematically described as follows:

(1) $$Z\left(\omega \right)=\frac{\tilde{V}}{\tilde{I}}=\left|\frac{\tilde{V}\left(\omega \right)}{\tilde{I}\left(\omega \right)}\right|\left(\cos\phi \left(\omega \right)+j\;\sin\phi \left(\omega \right)\right)=Z_{r}+j\;Z_{j}$$

This technique measures electrochemical impedance by analyzing the alternating current (AC) response to a small‐amplitude sinusoidal potential applied across a defined frequency range. Using EIS, the interfacial modifications (e.g., changes in current or potential) are evaluated through a frequency‐dependent transfer function. In this equation, $\tilde{V}$ (voltage) and $\tilde{I}$ (current) are phasors representing the amplitude and phase of sinusoidal signals. The term *ϕ* (*ω*) denotes the phase angle between input and output signals. In Equation (1), ω is angular frequency can be defined as ω=2πf, and j is the imaginary number can be defined as $j=i^{2}$.

**FIGURE 3:**
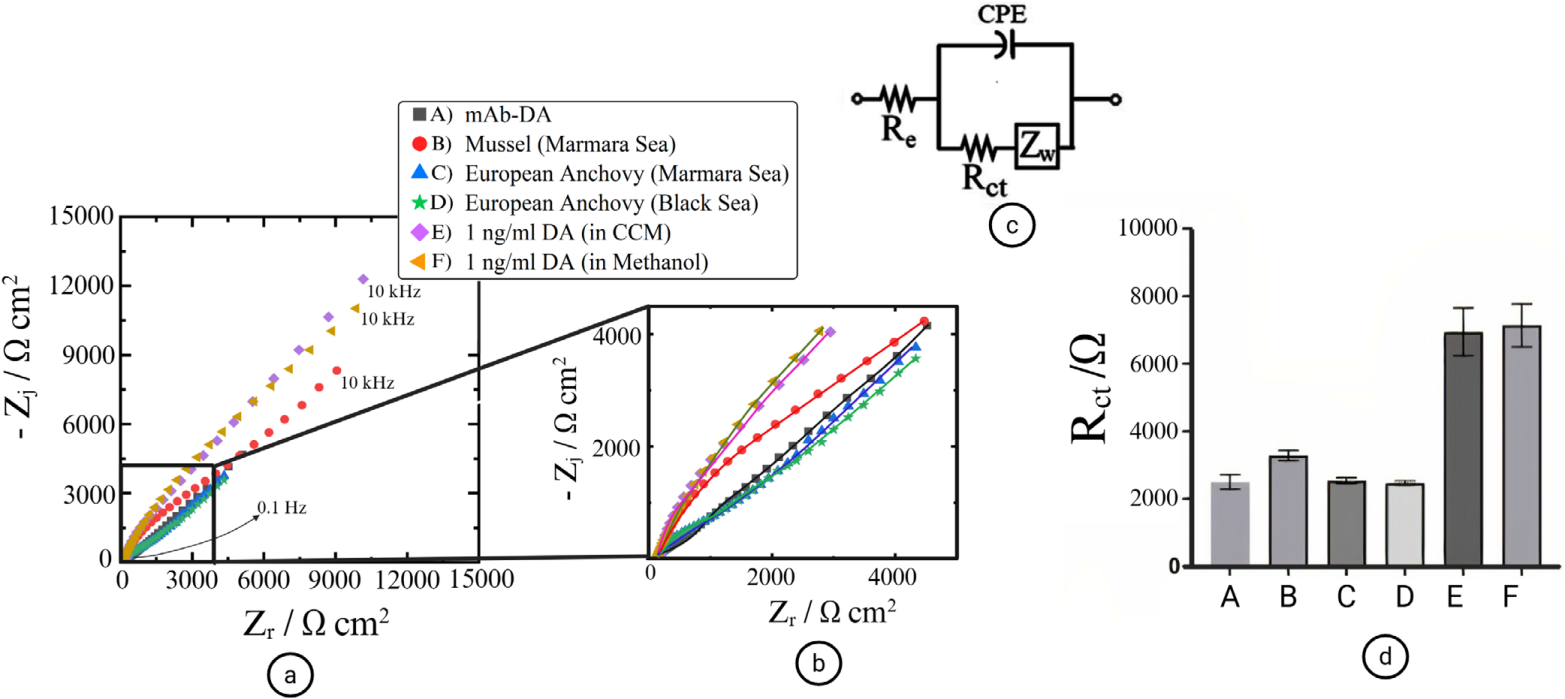
Nyquist plots of impedance responses from SPGEs for domoic acid detection in various seafood samples. (a) Complete impedance spectra across the entire frequency range, (b) magnified view of the high‐frequency region, (c) equivalent Randles circuit model used for data fitting, and (d) *R*_ct_ values obtained from EIS analysis. Electrochemical impedance spectroscopy (EIS) was performed in 1 mM [Fe(CN)_6_]^3−/4‐^ in 1 mM phosphate‐buffered saline (PBS), using a 10 mV AC amplitude at open circuit potential. All measurements were conducted at room temperature. Data fitting was carried out using Gamry Echem Analyst software. Each sample was tested in triplicate to ensure reproducibility, and error bars represent standard deviations. Created in BioRender. Avci, H. (2025) https://BioRender.com/ny0xgpb.

The model of the equivalent Randles circuit depicted in Figure 3c is optimized to model the impedance behavior of the electrochemical system. In this framework, the overall impedance Z, is formulated as (Koç et al. 2021b):

(2) $$Z=\;R_{e}+\frac{R_{\mathrm{ct}}+Z_{w}}{1+{\left(\mathrm{j\omega}\right)}^{\alpha }\left(R_{\mathrm{ct}}+Z_{w}\right)Q}$$

In Equation (2), *R*_e_ represents the electrolyte resistance, *R*_ct_ denotes the charge transfer resistance, *Z*_w_ corresponds to the Warburg impedance, constant phase element (CPE) exponent *α*, and its coefficient *Q*.

### 2.6 Scanning Electron Microscopy (SEM) Analysis of Antibody‐Immobilized SPGE Surfaces

Confirmation of the success of surface modifications was achieved by analyzing the working electrode surfaces of both bare SPGEs and bio‐immobilized SPGE surfaces using Field Emission Scanning Electron Microscopy (FESEM) to assess changes in surface morphology. Prior to SEM analysis, the electrodes were stored in a desiccator for at least 24 h. Subsequently, they were mounted onto aluminum stubs using carbon adhesive tape, and the working electrode regions of both bare SPGEs and bio‐immobilized SPGEs were imaged in secondary electron mode at 10 kV using a Hitachi Regulus 8230 Field Emission Scanning Electron Microscope (FESEM) (Figure 2i,ii).

### 2.7 Human Amniotic Fluid‐Derived Mesenchymal Stem Cells (hAF‐MSCs) Isolation and Culture

Mesenchymal stem cells (MSCs) were isolated from human amniotic fluid, which is a medical waste of patients whose consent was obtained with the signature of the patient's consent form according to the permission (80558721/170‐07; 22.06.2016) from the Clinical Research Ethics Committee of Eskisehir Osmangazi University. Human amniotic fluid‐derived MSCs (hAF‐MSCs) were characterized and cryopreserved at Cellular Therapy and Stem Cell Production Application and Research Centre, ESTEM, Eskişehir Osmangazi University.

The cryopreserved hAF‐MSCs vials were placed in a water bath at 37°C for a few seconds, the cells were resuspended in fresh medium in a conical‐bottom centrifuge tube and centrifuged at 200 × g for 10 min. The cells were washed three times and resuspended in 1 mL complete DMEM medium containing 10% FBS (Biological Industries, Beit Haemek, Israel), 0.2% primocin (Invivogen, San Diego, USA), and 1% (v/v) Glutamax (Sigma‐Aldrich, Burlington, USA). The cells were counted using trypan blue stain (Sigma‐Aldrich) and cell viability was calculated. The cells were cultured in 75 cm^2^ flasks at a density of 0.5 × 10^6^ cell/flask, at 37°C, in an atmosphere containing 5% O_2_ and 5% CO_2_. The cells were checked under a microscope every day, and the medium was changed every 3 days (Gaggi et al. 2022).

### 2.8 Flow Cytometric Analysis of hAF‐MSCs

Mesenchymal stem cell‐surface markers and viability were identified for hAF‐MSCs by flow cytometry to confirm that the cryopreserved cells are maintaining their characteristics. hAF‐MSCs attached to the surface of the culture dish were removed with 0.25% trypsin–EDTA (Biological Industries, Beit Haemek, Israel) and transferred to tubes containing culture medium and centrifuged. After centrifugation, the pellets were suspended and counted. 1 × 10^6^ cells/mL were suspended in its medium. Cells are then treated and incubated for 45 min at room temperature (dark) with fluorescein isothiocynate (FITC)‐conjugated monoclonal antibodies specific to CD90, CD73, CD44, and MHC‐I (HLA‐ABC), and phycoerythrin (PE)‐conjugated monoclonal antibodies specific to CD25, and MHC‐II (HLA‐DR), and Allophycocyanin (APC)‐conjugated monoclonal antibodies specific to CD45 cells surface markers. All the antibodies were obtained from BioLegend (San Diego, USA). After the washing step, the pellets were resuspended in 400 μL of the washing solution and were read by the Novocyte D3005 standard configuration (Agilent, Santa Clara, USA) instrument. Analysis was done using the NovoExpress flow cytometry software (version 1.5.0) (Agilent) (Gaggi et al. 2022).

### 2.9 SH‐SY5Y Cell Line Culture

Human neuroblastoma cells (SH‐SY5Y) were purchased from American Type Culture Collection (ATCC, Washington DC, USA). SH‐SY5Y cells were maintained in RPMI 1640 (Capricorn Scientific, Ebsdorfergrund, Germany) medium supplemented with 10% fetal bovine serum (FBS; Invitrogen/GIBCO, Grand Island, NY, USA), 1% (mL/mL) Glutamax (Sigma‐Aldrich, St Louis, MO, USA), and 1% penicillin‐streptomycin (Sigma‐Aldrich, St Louis, MO, USA), and the cells were cultured at 37°C in a humidified atmosphere of 5% CO_2_ and 95% air.

### 2.10 Cell Viability Assays

The DA concentrations employed to investigate cellular effects (0.1–100 μg/mL) were determined based on the following criteria: (i) the electrochemical biosensor detection limit (0.59 ng/mL, approximating 0.5 ng/mL), (ii) the TDI established by the FDA (75 ng/mL) (FDA 1993), and (iii) elevated DA concentrations to evaluate cellular responses. This tiered experimental approach was designed to holistically evaluate the biological effects of DA by incorporating doses spanning regulatory standards, biosensor detection capabilities, and thresholds associated with acute exposure. MTT assay was used to evaluate the cytotoxic activity of the DA and the elevated DA concentrations to be used in the experiments on hAF‐MSC and SH‐SY5Y cells. Cells were seeded at 5 × 10^3^ cells/well on the 96 well plates and incubated for 24 h at 37°C under constant humidity and 5% CO_2_. The cells were treated with 0.1, 0.5, 1, 50, 75, and 100 ng/mL and 10, 20, 30, and 100 μg/mL concentrations of the DA for 24 and 48 h at 37°C under constant humidity and 5% CO_2_. After that, 10 μL of MTT (Sigma‐Aldrich, St Louis, MO, USA) at a concentration of 5 mg/mL in PBS (Capricorn Scientific, Ebsdorfergrund, Germany) was added to the cultures, which were incubated for 2 h under darkness at 37°C and 5% CO_2_. Next, the medium was removed and 100 μL of DMSO (Sigma‐Aldrich, St Louis, MO, USA) was added to dissolve the resulting formazan salts. After 5 min, the product concentration was measured in a microplate reader (BIOTEK ELx808IU, Vermont, USA) at a wavelength of 570 nm. A total of three independent biological assays were performed using the same experimental conditions.

### 2.11 Quantitative Reverse Transcription PCR (RT‐qPCR)

After hAF‐MSC and SH‐SY5Y cells were incubated with 0.5 ng/mL, 75 ng/mL, 20 μg/mL, and 100 μg/mL concentrations of DA for the most effective time as 48 h, total RNA was isolated using GeneAll Hybrid‐RTM (GeneAll, Seoul, Korea) total RNA isolation kit. The RNA concentrations were measured using the Nanodrop spectrophotometer (ND‐2000, ThermoFisher Scientific, Waltham, MA USA) and then cDNA was synthesized using the ABT cDNA synthesis kit (Atlas Biotechnology, Ankara, Turkey). The PCR reaction was set using the AMPLIFYME SG Universal Mix kit (BLIRT, Gdańsk, Poland); the primer sequences are shown in Table 1. All procedures were performed according to the kit's manufacturer's instructions. The relative gene expression between the DA treated hAF‐MSC and the control (non‐treated hAF‐MSC) was done using the 2^−ΔΔCT^ method.

**TABLE 1:** List of the sequences of the primers that were used for the RT‐qPCR reaction in this study.

Gene name	The sequences of the primers
GRIA2	Forward: 5′‐TGC ATA CCT CTA TGA CAG TGA CA‐3′
	Reverse: 5′‐AGA ATT ACA CGC CGT TCC TTT‐3′
PRKDC (Koç et al. 2021b)	Forward: 5′‐CAC CTC TCT GAG GCT GTG C‐3′
	Reverse: 5′‐CGT CAT GTA AGC ATC AAT CAC C‐3′
MRE11	Forward: 5′‐ACA ACC TGC AAG CTC AGT GG‐3′
	Reverse: 5′‐TTA ATA CGC AGC AAA CCA ACA‐3′
ATM	Forward: 5′‐TGA TAG TAG TGT TAG TGA TGC AAA CG‐3′
	Reverse: 5′‐CAG CTA AAG GAT TAA TGG CAC CT‐3′
BRCA1	Forward: 5′‐ATC ATT CAC CCT TGG CAC A‐3′
	Reverse: 5′‐CAT GGA AGC CAT TGT CCT CT‐3′
BRCA2	Forward: 5′‐CCT GAT GCC TGT ACA CCT CTT‐3′
	Reverse: 5′‐GCA GGC CGA GTA CTG TTA GC‐3′
XRCC4	Forward: 5′‐CTT GGG ACA GAA CCT AAA ATG G‐3′
	Reverse: 5′‐GAC GTC TCA GGT AGT GAA GAA TCA‐3′
KU80	Forward: 5′‐CCC AAA TCC TCG ATT TCA GA‐3′
	Reverse: 5′‐CCC GGG GAT GTA AAG CTC‐3′
RAD51	Forward: 5′‐TGA GGG TAC CTT TAG GCC AGA‐3′
	Reverse: 5′‐CAC TGC CAG AGA GAC CAT ACC‐3′
KU70	Forward: 5′‐AGA GTG AAG ATG AGT TGA CAC CTT T‐3′
	Reverse: 5′‐CCA AGA GAT CTC GAT CAC TGC T‐3′
BCL2 (Trainer and Hardy 2015)	Forward: 5′‐TTG ACA GAG GAT CAT GCT GTA CTT‐3′
	Reverse: 5′‐ATC TTT ATT TCA TGA GGC ACG TT‐3′
ABCG2	Forward: 5′‐TT C ACG ATA TGG ATT TAC GG‐3′
	Reverse: 5′‐GTT TCC TGT TGC ATT GAG TCC‐3′
CYCS (Vivier and Orazem 2022)	Forward: 5′‐TGT GCC AGC GAC TAA AAA GA‐3′
	Reverse: 5′‐CCT CCC TTT TCA ACG GTG T‐3′
TNFA	Forward: 5′‐CAG CCT CTT CTC CTT CCT GAT‐3′
	Reverse: 5′‐GCC AGA GGG CTG ATT AGA GA‐3′
GAPDH	Forward: 5′‐GCA CAA GAG GAA GAG AGA GAC C‐3′
	Reverse: 5′‐AGG GGA GAT TCA GTG TGG TG‐3′

### 2.12 Statistical Analysis

Three independent experiments were conducted at different times for all experiments. Results are expressed as mean ± SE. SPSS 20.0 was used for statistical analysis. Data were analyzed using one‐way ANOVA‐Tukey's post hoc and paired *t*‐test. Differences between experimental and control groups were considered statistically significant when *p* < 0.05.

## 3 Results

### 3.1 Toxin Detection and Analysis

In Figure 3, the presence of domoic acid in marine sea food was investigated by the EIS method using SPGE, and the Nyquist graph (Figure 3a) was obtained which was modeled using the Gamry Echem Analyst program using the appropriate equivalent Randles circuit (Figure 3b). The Randles circuit model is suitable for modeling the kinetic and diffusion processes at the electrode/solution interface (Koç and Avci 2024; Koç et al. 2021a). Domoic acid was determined with the *R*_ct_ values (Figure 3c) obtained because of modeling. *R*_ct_ takes into consideration the transfer of ions from the electrolyte to the electrode and any changes that occur in the electrode's surface layers. The *R*_ct_ component in this model allows to quantitatively evaluate the binding efficiency of DA to the electrode surface (Jansson and Åkerman 2014; Ravalli et al. 2013). In experiments repeated at least three times, DA determination was performed by incubating the prepared stock mussels (Marmara Sea), European anchovy (Marmara Sea), and European anchovy (Black Sea) extractions on the electrode. When solutions containing 1 ng/mL DA in methanol and 1 ng/mL DA in CCM were applied to the immobilized electrode, these results confirmed that the biosensor was functioning properly. Afterwards, when the solutions containing the extraction of marine creatures were sent onto the biosensor, an increase in *R*_ct_ was observed only in mussels (Marmara Sea), meaning that the sample was determined to contain DA. When European anchovy (Marmara Sea) and European anchovy (Black Sea) extraction solutions were sent to the biosensor, DA could not be detected, and the presence of DA was not observed. The fact that *R*_ct_ values did not change in European anchovy (Marmara Sea) and European anchovy (Black Sea) extraction solutions supports that there is no specific interaction with DA. To determine the amount of DA in the mussel (Marmara Sea) sample, in which the presence of DA was detected with the increase in *R*_ct_, the calibration curve of the biosensor (Koç et al. 2021a), which was developed using the same electrode and immobilization method in our previous publication (Koç et al. 2021a), was used. Using the biosensor developed in our previous study (Koç et al. 2021a) under identical conditions (electrode type, immobilization method, electrochemical techniques, and environmental parameters), DA was detected at a concentration of 0.589635 ng/mL in mussels from the Marmara Sea region. Moreover, as observed in the Nyquist plot (Figure 3a), the biosensor successfully detected 1 ng/mL DA (in CCM). The CCM (cell culture medium) is a solution containing bovine serum albumin (BSA), amino acids, hormones, serum, and similar components. The selectivity of our biosensor toward DA in such a complex medium was further confirmed by analyzing the *R*_ct_ values in Figure 3d. As a result, although domoic acid was found in trace amounts in mussels obtained from the Marmara region, it was not found in anchovies obtained from both Marmara Sea and the Black Sea regions. This result is consistent with the literature that mussels tend to accumulate neurotoxins such as DA due to their position in the food chain and filtration properties (Bernstein et al. 2021). The absence of DA in anchovies can be explained by the metabolic characteristics of the species or low levels of exposure to DA. In conclusion, it demonstrates the presence of trace levels of DA in Marmara mussels, while proving the rapid and specific detection potential of the biosensor.

### 3.2 Cell Viability Assay Results

**FIGURE 4:**
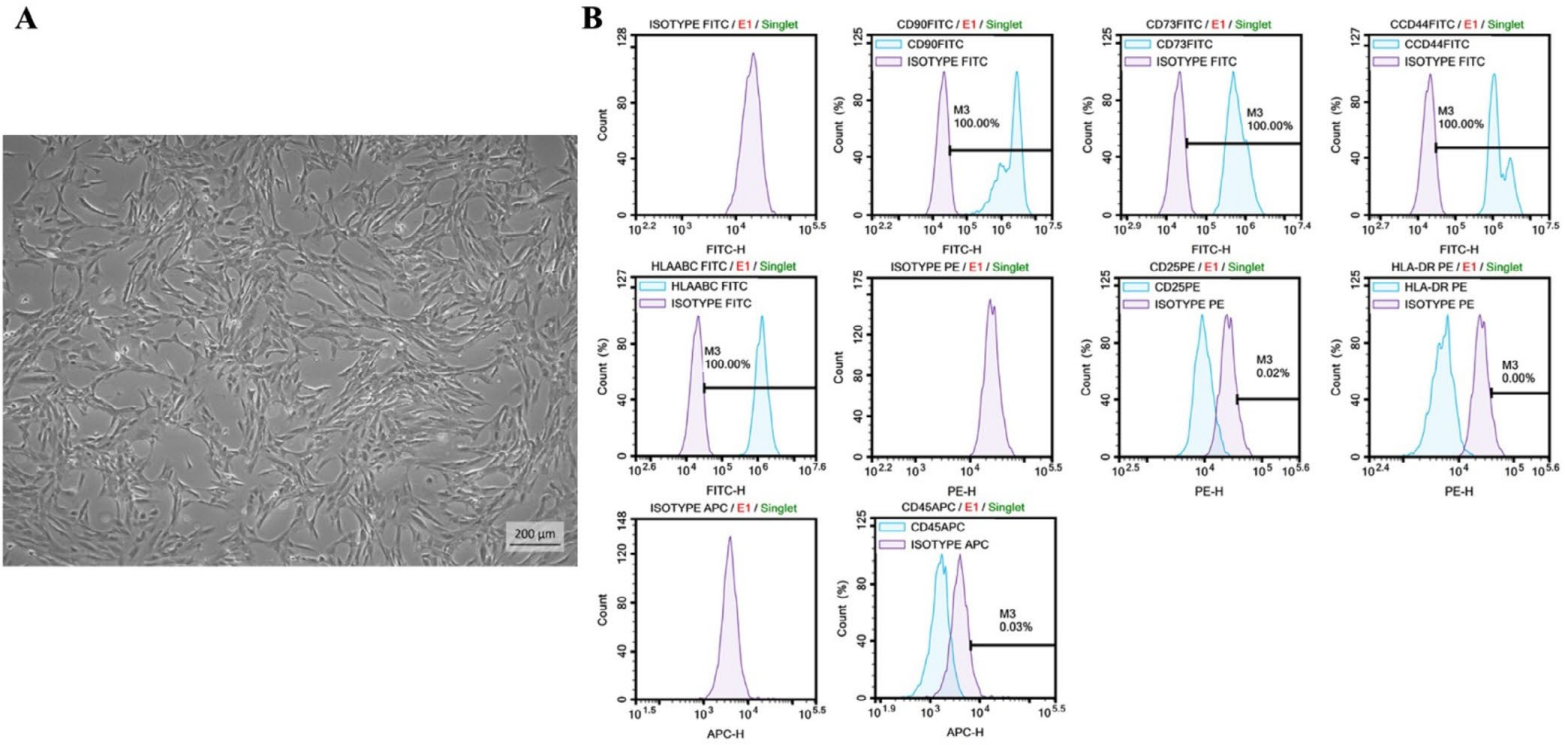
(A) Phase‐contrast microscope image of hAF‐MSCs at P3‐5 days (scale bar = 200 μm). (B) Flow cytometry results were performed for hAF‐MSCs‐specific surface markers, CD90 (100%), CD73 (100%), CD44 (100%), and MHC‐I (HLA‐ABC) (100%) were positive, and 0.02%, MHC‐II (HLA‐DR) (0%), and CD45 (0.03%) were negative. On the histogram, target antibodies are shown in blue and isotype antibodies (negative control) are shown in purple.

The cryopreserved vials containing the MSCs were thawed and cultured successfully, and the viability of the cells was 98%. The cultured cells were examined daily with phase‐contrast microscopy; the cells had fibroblast‐like, spindle, and star‐shaped morphology (Figure 4A).

The characterization of hAF‐MSCs was done by examining the expression of their surface markers using flow cytometry. hAF‐MSCs were found to be positive for CD90 (100%), CD73 (100%), CD44 (100%), and MHC‐I (HLA‐ABC) (100%), and negative for CD25 (0.02%), MHC‐II (HLA‐DR) (0%), and CD45 (0.03%). The results correspond to the criteria proposed by the International Society for Cellular Therapy (ISCT) (Gaggi et al. 2022; Figure 4B).

**FIGURE 5:**
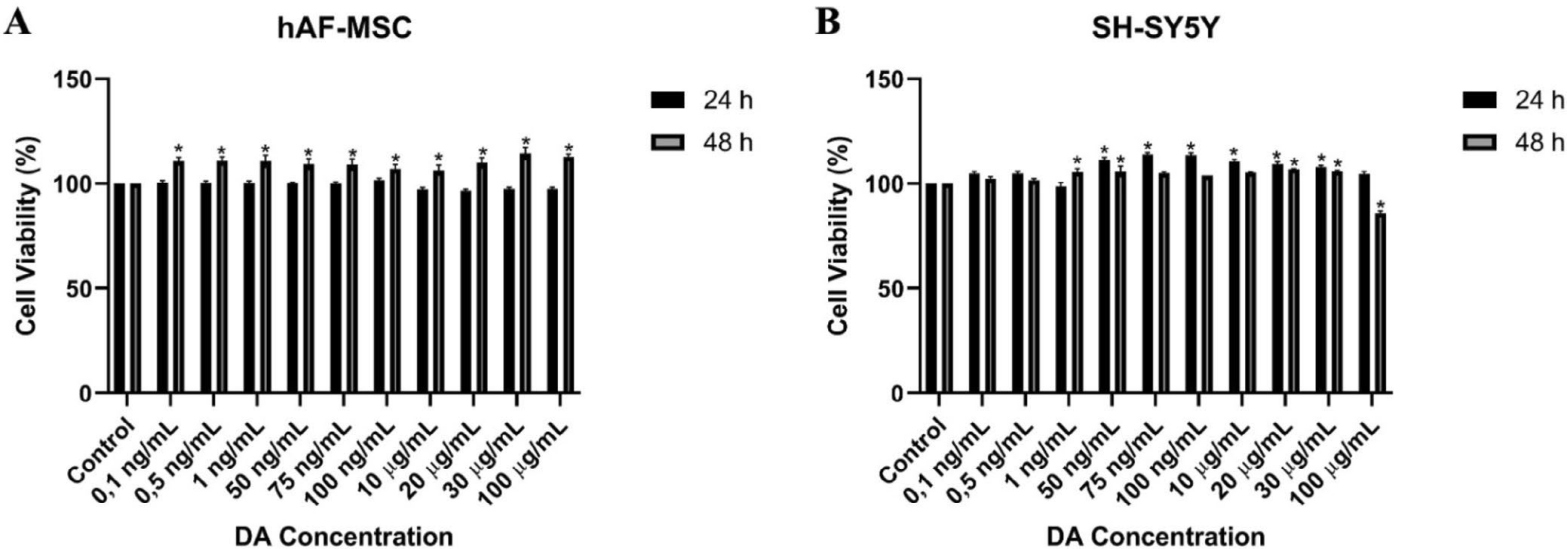
The effect of the application of DA at different concentrations on hAF‐MSC (A) and SH‐SY5Y cells (B). MTT test was performed at 24 and 48 h. The represented data are shown as the mean ± SE. (*) Indicates significant difference from the control group by the one‐way ANOVA‐Tukey's post hoc test (*p* < 0.05).

MTT results showed that there was no significant cytotoxic effect of the DA on hAF‐MSC and SH‐SY5Y cells at the end of the 48 h (Figure 5). At all concentrations applied to the MSC cells for 48 h, the cell viability increased by 1%–5% compared to control. At 100 μg/mL concentration of DA applied to the SH‐SY5Y cells, the cells' viability showed a decrease in comparison with the control; cell viability result was about 85%.

### 3.3 RT‐qPCR Analyses Results

To examine the gene expressions which are related to a cellular response against a cytotoxic agent and the DNA repair mechanism, the relative gene expression of PRKDC, MRE11, ATM, XRCC4, BRCA1, BRCA2, Ku80, Rad 51, Ku70, BCL2, ABCG2, CYCS, TNFA, and the glutamate receptor GRIA2 genes was analyzed. DA concentrations were determined based on the following: 100 μg/mL based on the MTT analysis result, 20 μg/mL the maximum concentration determined by the Washington Department of Fish and Wildlife (n.d.), 75 ng/mL based on the TDI of domoic acid for humans (Tenorio et al. 2021), and about 0.5 ng/mL based on the concentration which was detected by the biosensor.

**FIGURE 6:**
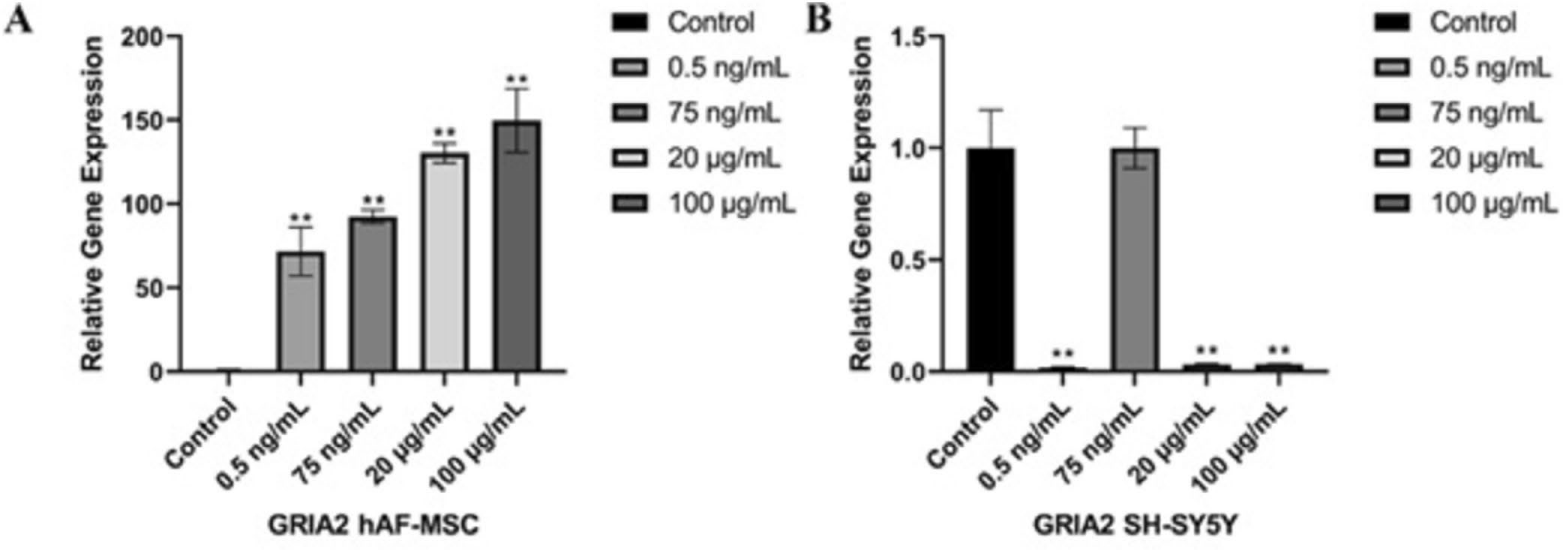
The gene expression results of the glutamate receptor GRIA2. (A) hAF‐MSCs. (B) SH‐SY5Y cells. Cells were treated with various concentrations of domoic acid for 48 h (0.5 ng/mL, 75 ng/mL, 20 μg/mL, and 100 μg/mL), relatively to their expression in the control (untreated cells) (*n* = 3, mean ± SD, **p < 0.01).

After hAF‐MSCs and SH‐SY5Y cells incubated with 0.5 ng/mL, 75 ng/mL, 20 μg/mL, and 100 μg/mL concentrations of DA for 48 h, the relative gene expression results of GRIA2 in hAF‐MSCs were statistically significantly upregulated; the higher the concentration, the greater the expression increased compared to their expression in the control group (the non‐treated cells) (Figure 6A). About the results of GRIA2 in SH‐SY5Y cells, the relative gene expression was statistically significantly downregulated at 0.5 ng/mL, 20 μg/mL, and 100 μg/mL concentrations of DA compared to their expression in the control group (the non‐treated cells) (Figure 6B).

**FIGURE 7:**
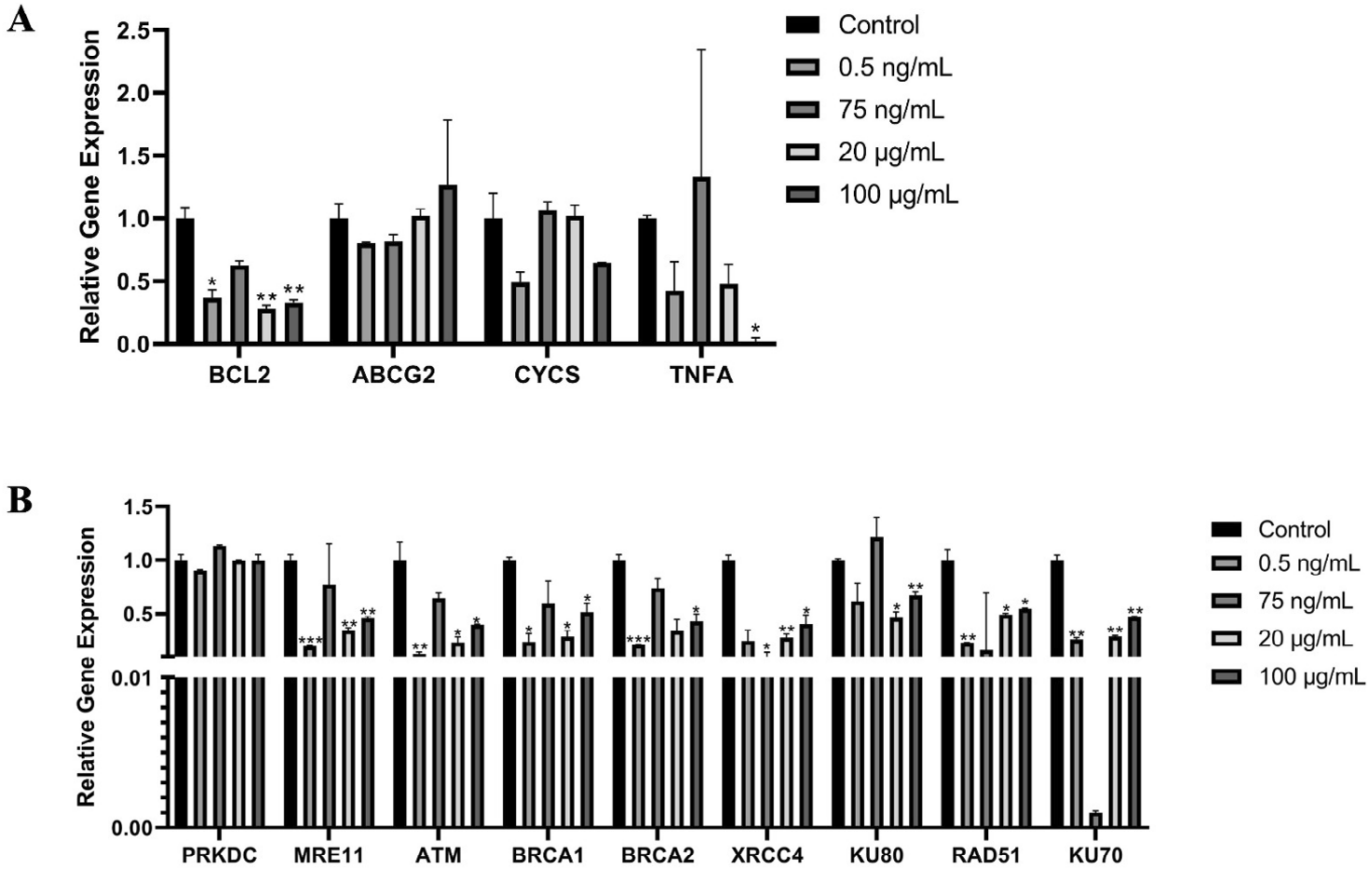
The gene expression results of hAF‐MSCs treated with various concentrations of domoic acid for 48 h (0.5 ng/mL, 75 ng/mL, 20 μg/mL, and 100 μg/mL) have been compared to their expression in the control (untreated MSCs). (A) The gene expression results of BCL2, ABCG2, CYCS, TNFA genes that are related to a cellular response against a cytotoxic agent. (B) The gene expression results of PRKDC, MRE11, ATM, XRCC4, BRCA1, BRCA2, Ku80, Rad 51, Ku70 genes that are related to the DNA repair mechanism (*n* = 3, mean ± SD, *p < 0.05, **p < 0.01, ***p < 0.001).

After hAF‐MSCs were incubated with 0.5 ng/mL, 75 ng/mL, 20 μg/mL, and 100 μg/mL concentrations of DA for 48 h, the relative gene expression results of MRE11, ATM, BRCA1, BRCA2, KU70, Rad 51, and BCL2 genes were statistically significantly downregulated compared to their expression in the control group (the non‐treated cells) at 0.5 ng/mL, 20 μg/mL, and 100 μg/mL concentrations of DA; the same result is applied for KU80 gene at 20 and 100 μg/mL concentrations of DA, and for XRCC4 gene at 75 ng/mL, 20 μg/mL, and 100 μg/mL concentrations of DA (Figure 7). While the gene expressions of PRKDC, ABCG2, and CYCS genes were statistically nonsignificant at all applied concentrations because the expression results were near to their expression in the control group (Figure 7).

**FIGURE 8:**
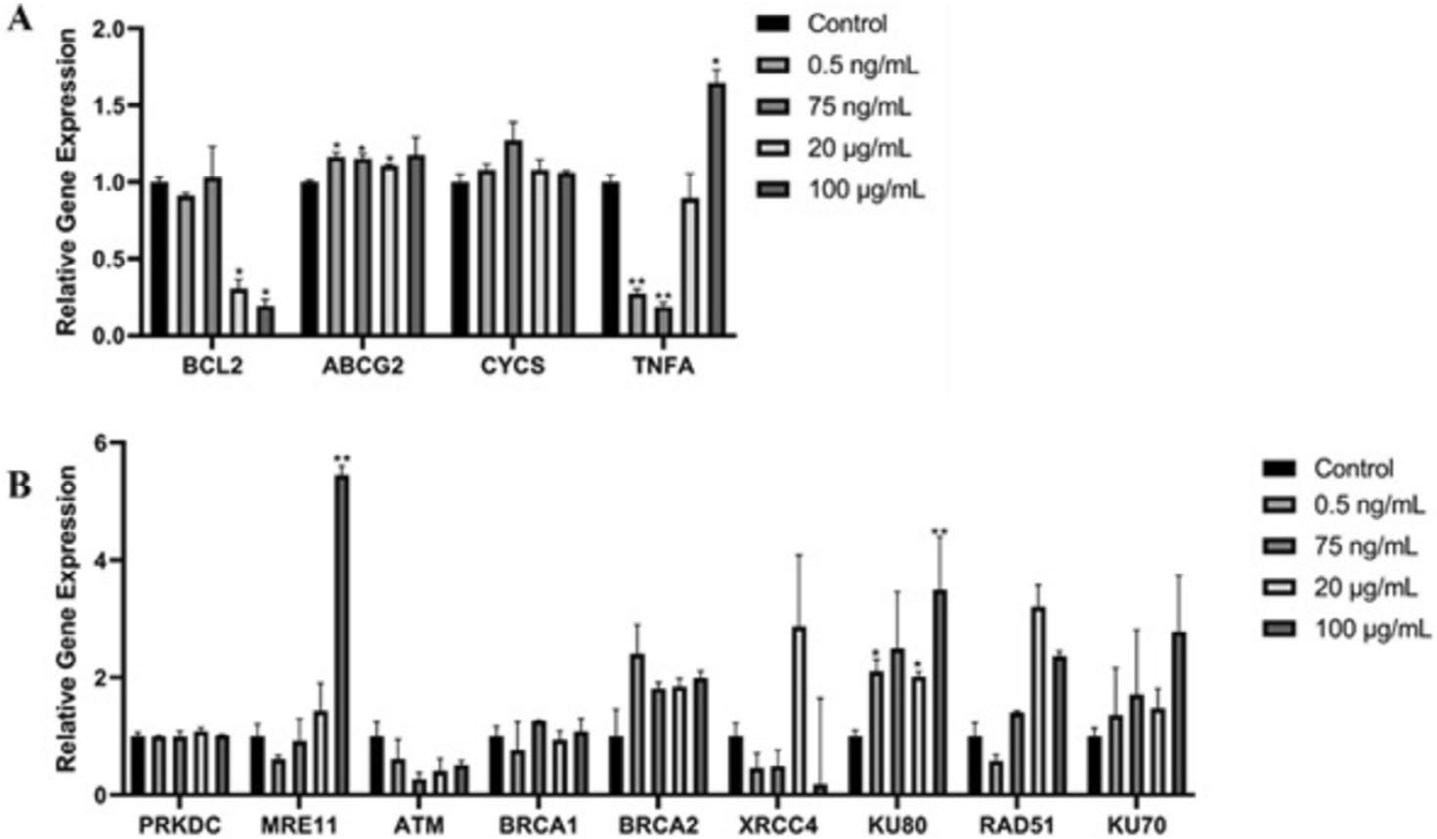
The gene expression results of SH‐SY5Y cells treated with various concentrations of domoic acid for 48 h (0.5 ng/mL, 75 ng/mL, 20 μg/mL, and 100 μg/mL) have been compared to their expression in the control (untreated SH‐SY5Y cells). (A) The gene expression results of BCL2, ABCG2, CYCS, TNFA genes that are related to a cellular response against a cytotoxic agent. (B) The gene expression results of PRKDC, MRE11, ATM, XRCC4, BRCA1, BRCA2, Ku80, Rad 51, Ku70 genes that are related to the DNA repair mechanism (*n* = 3, mean ± SD, *p < 0.05, **p < 0.01).

After and SH‐SY5Y cells incubated with 0.5 ng/mL, 75 ng/mL, 20 μg/mL, and 100 μg/mL concentrations of DA for 48 h, the relative gene expression results of PRKDC, ATM, BRCA1, BRCA2, XRCC4, Rad 51, KU70, CYCS, and TNFA were statistically nonsignificant at all applied concentrations, because the expression results were near to their expression in the control group (Figure 8B). While the gene expression of the KU80 gene at 0.5 ng/mL and 20 μg/mL concentrations of DA, and the MRE11 gene at 100 μg/mL concentrations of DA were statistically significantly upregulated relative to their expression in the control group. The same result applies for ABCG2 at 0.5 ng/mL, 75 ng/mL, and 20 μg/mL concentrations of DA, and for the TNFA gene at 100 μg/mL concentrations of DA. The BCL2 gene was statistically significantly downregulated compared to its expression in the control group (the non‐treated cells) at 20 and 100 μg/mL concentrations of DA, and the same result was for the TNFA gene at 0.5 and 75 ng/mL concentrations of DA (Figure 8A).

## 4 Discussion

The developed electrochemical biosensor was shown to be effective in detecting differential accumulation of DA as trace neurotoxin levels in marine species in the Marmara and Black Sea regions. The detection of DA exclusively in mussels from the Marmara Sea aligns with established ecological and physiological factors. Bivalves such as *Mytilus galloprovincialis* are filter feeders, continuously processing large volumes of water, which predisposes them to bioaccumulate toxins from harmful algal blooms (HABs) (Trainer and Hardy 2015; Dursun 2021). This behavior, combined with their position in the food chain, explains their higher susceptibility to DA contamination compared to pelagic fish like European anchovies, which exhibit transient exposure and efficient metabolic detoxification mechanisms (Lefebvre and Robertson 2010). The absence of DA in anchovies from both regions suggests either limited exposure to *Pseudo‐nitzschia* spp. (the primary DA‐producing algae) during the sampling period or species‐specific metabolic pathways that rapidly eliminate the toxin (Bernstein et al. 2021). EIS leverages its nondestructive nature, rapid response capability, and high sensitivity to transform an electrochemical system's response into a quantifiable signal, enabling precise analysis of dynamic changes at the electrode–solution interface (Koc et al. 2019; Ghorbanpoor et al. 2025; Avci et al. 2019; Crapnell and Banks 2024). Screen‐printed gold electrodes (SPGEs) are widely employed due to their cost‐effectiveness, reproducibility, and bio‐functionalization capacity. The integration of EIS with gold‐based SPGEs, as applied in this study, provides a rapid, label‐free, and highly sensitive platform for domoic acid detection (Madunić et al. 2022). The electrochemical biosensor demonstrated high specificity and sensitivity, detecting DA at trace concentrations of 0.589635 ng/mL in Marmara's mussels. The increase in *R*_ct_ upon DA binding underscores the biosensor's ability to capture antigen–antibody interactions at the electrode interface. The consistency of these results with prior calibration data from similar immobilization protocols reinforces the reproducibility of the method. Additionally, this study provides an integrated assessment of the biological effects of environmental contamination on human cells by testing a wide range of DA concentrations, ranging from levels detected by an electrochemical biosensor (0.5 ng/mL) to regulatory limits (75 ng/mL) and acute exposure ranges (20–100 μg/mL). This integrated approach aligns with recent trends in food and environmental safety research, where detection technologies are increasingly evaluated not only for analytical sensitivity but also for their biological relevance (Lefebvre and Robertson 2010; FDA 1993). The potential health impact of trace levels of marine toxins such as domoic acid cannot be understood solely through detection data; rather, cell‐based toxicity testing is essential to validate and contextualize exposure risks (Madunić et al. 2022; Gajski et al. 2020). Hence, our study provides a translational perspective linking biosensor results to functional cellular responses, which reflects a multidisciplinary approach increasingly emphasized in food safety and environmental toxicology frameworks (Tenorio et al. 2021; Petroff et al. 2021). In this study, the electrochemical biosensor developed herein was employed to investigate DA levels in various marine organisms, revealing trace concentrations (0.59 ng/mL) in mussels from the Marmara Sea. The concentrations identified by the biosensor as 0.5 ng/mL, along with higher doses of 75 ng/mL, 20 μg/mL, and 100 μg/mL, were directly applied to cytotoxicity assays to evaluate cellular responses at environmentally obtained and regulatory relevant doses, thereby enabling a comprehensive assessment of DA's biological impact.

On the other hand, domoic acid is an analog of glutamate; a neurotransmitter in the brain that activates glutamate receptors, and it has excitatory effects on nerve cells (Zhou and Danbolt 2014). In this study, various domoic acid concentrations were applied to amniotic fluid‐derived mesenchymal stem cells (hAF‐MSC) from the fetal period tissues and the human neuroblastoma cell line (SH‐SY5Y) as a model for neuron‐like cells to evaluate their cytotoxicity. As a result of the 48 h application, it increased proliferation in hAF‐MSC and decreased cell viability in SH‐SY5Y cells at a concentration of 100 μg/mL. Gajski et al. (2020) reported that adverse cytotoxic effects of DA in nontarget human peripheral blood cells (HPBCs). DA, which has a neurotoxic effect, is reported to have a cytotoxic effect in different cells (Madunić et al. 2022; Gajski et al. 2020; Hassoun et al. 2021). Our results showed that proliferation increased in mesenchymal stem cells after 48 h of application. DA was shown to have no cytotoxic effect on nontargeted hAF‐MSC and to promote cell proliferation. This increase in proliferation in MSCs is thought to be due to the neural differentiation potential of hAF‐MSCs (Gaggi et al. 2022; Jiménez‐Acosta et al. 2022; Xia et al. 2013; Vawda and Wilcox 2008), glutamate receptors are overexpressed after DA administration and thus proliferation increases.

The AMPA receptor is not directly involved in DNA repair mechanisms. However, overactivation of glutamate receptors can cause cellular damage such as oxidative stress and calcium imbalance. Such cellular stresses can lead to DNA damage and indirectly trigger DNA repair mechanisms. On the other hand, it has been demonstrated that the DA effects were completely prevented by the antagonist of the AMPA receptor (Jansson and Åkerman 2014; Hogberg and Bal‐Price 2011; Benke and Traynelis 2019; Wright and Vissel 2012). AMPA receptors are an important component of the ionotropic glutamate receptor family and the GRIA2 (Glutamate Ionotropic Receptor AMPA Type Subunit 2) gene encodes one of their subunits. GRIA2 plays a critical role in maintaining neuronal homeostasis by regulating the calcium permeability of the AMPA receptor. However, expression levels of the GRIA2 gene can alter the susceptibility to DNA damage in neuronal cells by directly affecting the calcium permeability of AMPA receptors (Wright and Vissel 2012) As the concentrations of DA used in the study increased, GRIA2 gene expression in hAF‐MSCs increased in a concentration‐dependent manner compared to the control group and the concentration of FDA‐determined TDI of domoic acid in SH‐SY5Y cells was similar to the control group but decreased at other concentrations, indicating that DA activates the receptor in hAF‐MSCs but not in the neuroblastoma cell line SH‐SY5Y. These findings provide important data for understanding the effect of DA in neurodegenerative processes and the role of GRIA2 in neuroblastoma cells.

To examine the gene expression of the genes that are related to cellular response against a cytotoxic agent, DA and the DNA repair mechanism, the relative gene expression of PRKDC, MRE11, ATM, XRCC4, BRCA1, BRCA2, Ku80, Rad 51, Ku70, BCL2, ABCG2, CYCS, TNFA were analyzed. After hAF‐MSCs were incubated with domoic acid (DA), the relative gene expression results of MRE11, ATM, BRCA1, BRCA2, Ku70, Ku80, XRCC4, Rad 51, PRKDC, and BCL2 genes showed that while some genes were like the control group, some genes were statistically significantly downregulated compared to the control group. An enhanced DNA repair capacity is generally observed in normal (pluripotent or multipotent) stem cells compared to differentiated cells (Frosina 2010). This suggests that stem cells tend to protect their genetic material with advanced DNA repair mechanisms. Our results suggest that DA in hAF‐MSCs DNA repair capacity is at a level that is not needed and does not cause damage to the cells in this respect; these findings are in parallel with the results of cell viability and proliferation (Bellagamba et al. 2016). Furthermore, considering that the expression levels of ABCG2, which encodes a membrane transporter that transports drugs across the plasma membrane and plays a role in multidrug resistance (MDR) (Haider et al. 2015; Borst and Elferink 2002), and CYCS genes, which play a role in the initiation of the apoptosis process (Eleftheriadis et al. 2016), were not statistically significant at all applied concentrations, the results obtained suggest that DA in hAF‐MSCs is at a level that does not require DNA repair capacity and does not damage the cells in this respect; these findings are in parallel with the results of cell viability and proliferation (Frosina 2010; Bellagamba et al. 2016; Vinoth et al. 2008). This can be explained not only by the general resistance of MSCs, attributing to their potential to form teratomas, but also by their developed defense mechanisms due to their fetal origin (Bleyl and Schoenwolf 2018; Gill and Kumara 2019).

SH‐SY5Y cells were incubated with domoic acid (DA) at concentrations of 0.5 ng/mL, 75 ng/mL, 20 μg/mL, and 100 μg/mL for 48 h. The relative gene expression results of PRKDC, ATM, BRCA1, BRCA2, XRCC4, Rad 51, KU70, CYCS, and TNFA genes were not found to be statistically significant at all applied concentrations. On the other hand, the gene expression of KU80 and MRE11 genes, which play a role in the DNA repair mechanism (Radad et al. 2018), was statistically significantly upregulated compared to the control group. This situation showed that DA concentrations applied to neural‐like cells could not maintain their genetic stability. Moreover, the statistically significant downregulation of TNFA and BCL2 genes suggests that the antiapoptotic properties of neural‐like cells, which show genetic instability as revealed by the KU80 gene increase, are weakened by the DA effect (Lu et al. 2013). On the contrary, the statistically significant upregulation of the ABCG2 gene in healthy neural‐like cells indicates that the applied DA concentrations may trigger an adaptive resistance to toxic effects. ABCG2, a membrane‐bound efflux transporter and a major component of the blood–brain barrier, plays a critical role in cellular detoxification and neuroprotection by actively exporting xenobiotics and reducing intracellular oxidative stress. In the context of this study, the elevated expression of ABCG2 may represent an intrinsic protective mechanism activated by SH‐SY5Y cells to maintain genomic stability under DA‐induced stress. Notably, this response appears to be sufficient to prevent the upregulation of other DNA repair genes, suggesting that ABCG2‐mediated detoxification might alleviate the cellular need for further DNA repair activation. These findings align with the established role of ABCG2 in multidrug resistance and neuroprotection, emphasizing its potential contribution to cellular resilience in neurotoxic environments (Breedveld et al. 2005).

For SH‐SY5Y cells, the cells' viability showed a slight decrease in comparison with the control at 100 μg/mL of DA. About the expression results of the glutamate receptor, the increase of the GRIA2 gene shows that the DA activates the receptor in hAF‐MSCs but not in the neuroblastoma cell line, SH‐SY5Y. It is thought that in hAF‐MSCs, genetic stability is preserved in parallel with the increase in GRIA2 gene expression level depending on the increase in DA concentration, and accordingly there is no change in DNA repair capacity, and similarly, it has no effect on hAF‐MSC viability and proliferation.

This study focuses on developing a high‐sensitivity electrochemical biosensor system to detect domoic acid (DA), a neurotoxin found in seafood. Additionally, it examines the cytotoxic, neurotoxic, and DNA repair‐related effects of DA at the FDA's highest permitted concentration and the levels identified in this study. The findings reveal that DA influenced gene expression related to cytotoxic response, DNA repair, and neurotoxicity in hAF‐MSC and SH‐SY5Y cells but did not significantly impact cell viability within 48 h. While hAF‐MSCs showed no major signs of cellular damage, SH‐SY5Y cells exhibited a slight reduction in viability at high concentrations. Notably, DA modulated glutamate receptor (GRIA2) expression in hAF‐MSCs but not in SH‐SY5Y cells, suggesting differential neuronal responses. SH‐SY5Y cells also exhibited an upregulation of DNA repair and membrane transporter genes (e.g., ABCG2), indicating an adaptive resistance mechanism. The biosensor's detection of 0.59 ng/mL DA in Marmara mussels informed the design of low‐dose experiments (0.5–75 ng/mL), wherein changes in glutamate receptor activity and DNA repair mechanisms in cells were examined in detail. High doses (20–100 μg/mL) triggered adaptive resistance mechanisms in neuroblastoma cells, such as an increase in gene expression, indicating a change in their capacity to cope with DA‐induced stress. This finding is consistent with previous studies highlighting ABCG2's role in protecting neuronal cells by facilitating the efflux of toxic agents and maintaining cellular homeostasis, particularly under neurotoxic stress conditions (Haider et al. 2015).

In this study, a highly sensitive electrochemical biosensor system was successfully developed for the investigation of DA in seafood. In addition, when MTT assay was performed at various doses to determine the IC50 dose in hAF‐MSC and SH‐SY5Y cells, the IC50 concentration could not be determined when the FDA‐determined TDI of domoic acid concentrations of 0.1 ng/mL and 100 μg/mL were applied because cell viability decreased by at most 10% at these applied concentrations. Among these concentrations, when the FDA‐determined TDI of domoic acid concentrations selected (75 ng/mL), 0.5 ng/mL, 20 μg/mL, and 100 μg/mL were applied, the expressions of DNA repair mechanism genes, drug resistance genes, apoptotic and antiapoptotic genes analyzed in hAF‐MSCs were like the control group. As a result, it was determined that hAF‐MSC, which are cells belonging to the fetal period, were not damaged by the neurotoxic, cytotoxic, apoptotic, and genotoxic effects of DA at the concentrations applied in this study, including the limit determined by the FDA, and these findings were also supported by the results showing that these cells did not affect the viability and proliferation. On the other hand, in SH‐SY5Y cells, there was a statistically significant increase in DNA repair mechanism genes, MRE11 and KU80, while the expression of the antiapoptotic gene BCL‐2 decreased, and an increase in the expression of the apoptotic TNFA gene was observed. However, we believe that this apoptotic effect should be supported by functional and protein expression analyses. Although Western blotting for KU80 and MRE11 proteins was not included in the current study, future research will aim to validate these transcriptomic findings at the protein level. While the gene expression level of GRIA2 subunit, which is a member of AMPA receptor family and negatively associated with calcium‐mediated oxidative stress, did not change in hAF‐MSCs, in SH‐SY5Y cells, it was similar to the control group only at the FDA‐determined TDI of domoic acid concentration, while its decrease at other concentrations was found to be correlated with oxidative stress‐induced DNA damage and increased expression of MRE11 and KU80 genes and a slight decrease in cell viability.

In conclusion, it is a remarkable finding that the neurotoxin DA did not have a significant cytotoxic effect on hAF‐MSCs and SH‐SY5Y cells at the concentrations applied to them for 48 h but had some toxic effects on SH‐SY5Y cells. The SH‐SY5Y cells exhibited protection against DA, indicating the cancer cells are enhancing their DNA repair mechanisms to protect themselves against apoptosis and cell death. It is also supported by the upregulation of genes related to DNA repair and membrane transporter gene ABCG2. However, these analyses at the genotypic level should be supported by analyses determining phosphorylated protein expressions in the future.

This integrated approach aligns with recent trends in food and environmental safety research, where detection technologies are increasingly evaluated not only for analytical sensitivity but also for their biological relevance (Lefebvre and Robertson 2010; Petroff et al. 2021). The potential health impact of trace levels of marine toxins such as domoic acid cannot be understood solely through detection data; rather, cell‐based toxicity testing is essential to validate and contextualize exposure risks (Madunić et al. 2022; Gajski et al. 2020). Hence, this study provides a translational perspective, linking biosensor data with functional cellular outcomes, particularly focusing on DNA repair, apoptosis, and neurotoxicity‐related pathways in stem and neuronal cell models. Such a combination of analytical chemistry and toxicogenomics reflects a multidisciplinary paradigm that is encouraged in food safety and environmental toxicology frameworks (Tenorio et al. 2021). Altogether, this study contributes to a more comprehensive understanding of domoic acid's cellular effects across a concentration spectrum, offering valuable insight for seafood safety monitoring and risk assessment strategies.

## Author Contributions

**Emilia Qomi Ekenel:** data curation, formal analysis, writing – original draft, project administration. **Yucel Koc:** conceptualization, methodology, data curation, formal analysis, project administration. **Burcugul Altug:** data curation, formal analysis, methodology. **Merve Nur Soykan:** investigation, validation, visualization. **Bahar Demir Cevizlidere:** formal analysis, project administration, supervision. **Sibel Gunes Bagis:** resources, supervision, writing – original draft. **Onur Uysal:** conceptualization, resources, project administration, supervision, funding acquisition, writing – review and editing, writing – original draft. **Tugba Semerci Sevimli:** formal analysis, software. **Murat Sevimli:** formal analysis, software, funding acquisition. **Ayla Eker Sariboyaci:** conceptualization, methodology. **Huseyin Avci:** conceptualization, data curation, software, project administration, supervision, funding acquisition, writing – original draft. Dr. Onur Uysal served as a main, lead contributing author, with a primary role in manuscript preparation and revision.

## Funding

This study has been supported by Eskisehir Osmangazi University Scientific Research Projects Coordination Unit (ESOGU‐BAP) under grant number 201911061 and 201815044 and by The Scientific and Technological Research Council of Turkey (TUBITAK) under grant number 20AG031.

## Ethics Statement

This study was conducted with the approval of the Republic of Turkey Ministry of Agriculture and Forestry, General Directorate of Fisheries and Aquaculture (67852565‐140.03.03‐E.1415263, 08.06.2017) and Eskisehir Osmangazi University Clinical Research Ethics Committee (80558721/170‐07; 22.06.2016).

## Conflicts of Interest

The authors declare no conflicts of interest.

## Data Availability

The data that support the findings of this research are available from the corresponding author upon reasonable request.
